# Importance of an Interdisciplinary Approach in the Treatment of Women with Endometriosis and Chronic Pelvic Pain

**DOI:** 10.1055/s-0043-1777001

**Published:** 2023-11-29

**Authors:** Júlia Kefalás Troncon, Gabrielle Barbosa Anelli, Omero Benedicto Poli-Neto, Julio Cesar Rosa e Silva

**Affiliations:** 1 Departamento de Ginecologia e Obstetrícia da Faculdade de Medicina de Ribeirão Preto da Universidade de São Paulo, Ribeirão Preto, SP, Brazil

Endometriosis, from a pathophysiological standpoint, can be defined as active endometrium-like tissue outside the uterus. However, to better understand and approach this complex disease, it should be addressed as a chronic inflammatory disease that can cause pelvic pain during a long period of a women's reproductive lifetime. 1 Due to nociplasty, women affected by endometriosis can also maintain pain despite adequate organic treatment, especially considering that this disease has no objective cure. 1 2 On the other hand, chronic pelvic pain(CPP) can be defined as pain perceived in the lower abdomen, lasting for at least six months, that has a negative impact on quality of life and that demands treatment. 2 CPP may have a gynecologic etiology such as endometriosis, but most frequently has a multifactorial nature, involving gastrointestinal, urinary, psychological, and musculoskeletal systems.

Furthermore, women suffering from endometriosis frequently manifest other overlapping pain conditions such as irritable bowel syndrome, painful bladder syndrome, fibromyalgia, migraine headaches, among others. 3 This may be due to a lower pain threshold, cross-organ sensitization, 3 enhanced visceral pain or a common pathophysiological origin yet to be elucidated. Therefore, when managing pain in women suffering from endometriosis and/or CPP, an isolated gynecological approach will usually be insufficient.

For optimum care, the treatment must be patient centered, rather than disease centered. 4 So nowadays, more attention has been given in studies on the beneficial impact of an interdisciplinary approach toward CPP and endometriosis. Preferably, a true interdisciplinary team discusses together the best treatment for each individual patient, instead of a mere multidisciplinary setting where different health care professionals focus only in their own specific intervention. Quality of life assessment and improvement should be one of the main goals since a cure is most often unattainable.

An interdisciplinary team ideally would be composed by medical staff with specific training in this area of expertise, and by a nurse, psychologist, physical therapist, occupational therapist, nutritionist, and physical educator. The medical team should be composed of not only gynecologists, but also urologists, gastroenterologists, psychiatrists, and pain management specialist.

Patients with endometriosis and CPP commonly have associated mood disorders, with high levels of anxiety and depression. Up to 73% of anxiety and 40% of depression was the prevalence found in a cross sectional controlled study. 5 Notably, these women manifest a pain catastrophizing profile 3 6 that worsens the experiencing of the pain. So, a psychological approach is of upmost importance, as well as a program in pain education, 3 which will help the patient feel more responsible for her own improvement. Coping strategies and constructive attitudes can positively modulate the well-being of these women. A qualitative study showed that daily life attitudes can influence the experience of pain; the categories identified were: shaping life by pain, isolating from social contact, avoiding sexual relationship, seeking pain relief, and seeking positive strategies. 7

Endometriosis can also lead to dyspareunia, which is pain during sexual intercourse. This symptom can aggravate the negative impact of the disease in personal and loving relationships; up to 30% of women reported they frequently had to interrupt sexual intercourse because of pain, and 66% are afraid of pain before intercourse which can lead to avoidance of contact. 8 The prevalence of sexual distress was of 78% in one study, 8 and its origin multifactorial, associated with increased tone of the pelvic floor muscles, 9 due to a possible history of sexual, physical or moral abuse 10 or due to endometriosis itself.

In addition to a medical and psychological approach toward the sexual dysfunction and distress, the evaluation by a physical therapist is mandatory. Besides form dyspareunia, a physical therapist will also improve symptoms of myofascial syndrome which are common in women suffering from endometriosis and other causes of CPP, what might improve dyspareunia. Approximately 85% of women with CPP have musculoskeletal associated disorders; myofascial pain syndrome can be managed, among other strategies, by trigger point anesthetic injections, therapeutic ultrasound 11 and also by acupuncture. 12 Despite the lack of robust evidence, some studies show beneficial effects of acupuncture in the treatment of these conditions. 12 Physical exercise is another domain that should be addressed and encouraged. Even though without high-quality level of evidence, the practice of physical activity has almost no adverse effect, if there is no medical contraindication. Supposedly, the mechanism involved should be reduction of oxidative stress; regular practice of aerobic and other forms of physical activity apparently have a positive impact in diseases with an inflammatory background such as endometriosis. 13 In CPP in general, both aerobic and anaerobic activities seem to have an analgesic effect, conditioning hypoalgesia by pain modulation and effects on baroreceptors. 14 However, women with endometriosis apparently have altered mechanisms of central nociceptors, and also greater tendency to avoid the practice of physical activity. 14

Regarding the dietetic effects on endometriosis, a wide variety of data are available, even though with low grade of evidence. Nutritional aspects influence hormonal and inflammatory balance and as so have been implicated in the physiopathology of endometriosis. 15 There appears to be a protective impact on the consumption of dairy products and calcium and tryptofan, 16 probably through the induction of anti-oxidative mechanisms and improving general well-being. 15 16 Red meat and saturated fatty acids, on the other hand, appear to have a negative effect on the disease. 17

Consequently, dietary interventions seem to have a positive implication on the management of CPP and endometriosis. Especially when considering other associated diseases such as irritable bowel syndrome and painful bladder, for example, that could also benefit from dietary modifications. Nirgianakis et al. 18 suggest a symptom based approach in which patients with gastrointestinal related symptoms could mostly benefit from low-FODMAP (fermentable oligosaccharides, disaccharides, monosaccharides and polyols) and/or gluten-free diet, and a Mediterranean diet could be encouraged in general to improve global health. 18 Most importantly, nutritional changes could help patients to perceive how lifestyle habits can influence on their perceived pain, and how engaging in their own treatment is of great relevance toward improvement. 19

Finally, occupational therapists could help patients develop adaptative strategies when managing the burden that CPP has on almost all domains of social and professional aspects of their life. 20 Not only occupational activities, but also self-care, self-esteem and leisure are affected. Patients should benefit from the help of an occupational therapist in rehabilitation, and developing tools toward coping. 20

Endometriosis is a chronic disease that affects women in productive age and has significant economic impact. 21 CPP often accompanies endometriosis but also has other frequent etiologies, for this, questionnaires that assess quality of life and the involvement of other organs should be incorporated into the initial and ongoing evaluation of these patients as current practice.

Therefore, we recommend that an interdisciplinary approach would be of benefit in providing an optimum patient centered care.
